# Optimization of CRISPR/LbCas12a-mediated gene editing in *Arabidopsis*

**DOI:** 10.1371/journal.pone.0265114

**Published:** 2022-03-25

**Authors:** Qiang Zhang, Yan Zhang, Yiping Chai

**Affiliations:** 1 College of Plant Protection, Shandong Agricultural University, Tai’an, China; 2 Jiangsu Academy of Agricultural Sciences, Nanjing, Jiangsu, China; Oklahoma Medical Research Foundation, UNITED STATES

## Abstract

CRISPR/LbCas12a system (LbCpf1) has been widely used for genome modification including plant species. However, the efficiency of CRISPR/LbCas12a varied considerably in different plant species and tissues, and the editing efficiency needs to be further improved. In this study, we tried to improve the editing efficiency of CRISPR/LbCas12a in *Arabidopsis* by optimizing the crRNA expression strategies and Pol II promoters. Notably, the combination of tRNA-crRNA fusion strategy and RPS5A promoter in CRISPR/LbCas12a system has highest editing efficiency, while CRISPR/LbCas12a driven by EC1f-in(crR)p had the highest ratio of homozygous & bi‐allelic mutants. In addition, all homozygous & bi‐allelic mutants can be stably inherited to the next generation and have no phenotypic separation. In this study, the editing efficiency of the CRISPR/LbCas12a system was improved by selecting the optimal crRNA expression strategies and promoter of LbCas12a in *Arabidopsis*, which will prove useful for optimization of CRISPR/LbCas12a methods in other plants.

## Introduction

CRISPR/Cas has been applied to plant genome editing in a variety of plants, such as rice, wheat, tobacco, and *Arabidopsis* [1–3]. It is a powerful tool for improving agricultural character to meet market needs [4]. However, the editing induced by SpCas9 strictly requires a canonical NGG protospacer-adjacent motif (PAM), significantly limiting its scope of application. Cpf1 (CRISPR from *Prevotella* and *Francisella* 1) represents a new class 2/type V CRISPR RNA-guided endonuclease which recognizes the thymidine-rich protospacer-adjacent motif (PAM) to expand the range of RNA-guided genome editing [5]. The maturation of crRNA does not require the assistance of trans-activating crRNA (tracrRNA) and the length of crRNA (about 43 nt) is shorter than sgRNA of CRISPR/Cas9, making it suitable for multi-gene editing [6]. DNA cleavage occurs at the far end of the PAM and produces staggered ends with 5 nt overhangs, which may improve the efficiency of gene replacement [5]. Therefore, The CRISPR/Cpf1 system is considered to be a gene editing tool with promising applications. CRISPR/LbCas12a (LbCpf1) is the most commonly used CRISPR/Cpf1 system, which has been used to mediate targeted genome modification in mammalian cells and some plant species such as rice, *Arabidopsis*, soybean and tobacco [7–12]. The PAM requirement of CRISPR/LbCas12a is “TTTV”, which is advantageous for targeting promoters and other AT-rich sites in the gene coding region, and facilitates gene knockout and regulation. However, the editing efficiencies of CRISPR/LbCas12a system varied greatly between different plant species and tissues, and produced less homozygous mutant [13]. *Arabidopsis*, as an important model plant, has been widely used in the study of plant molecular genetics. However, there are few reports on the use of CRISPR/LbCas12a as a gene modification tool in *Arabidopsis*. It may be related to the low editing efficiency of CRISPR/LbCas12a under normal *Arabidopsis* growth temperature (e.g., 22°C) [13]. Optimizing conditions for its use within *Arabidopsis* is therefore needed to achieve highly efficient genome editing. The expression of sgRNA and Cas9 has been shown to influence the editing efficiency of the CRISPR/Cas9 system in previous studies [14–18]. Therefore, screening and testing combinations of different promoters of LbCas12a and crRNA expression strategies is an effective strategy to improve the editing efficiency of CRISPR/LbCas12a in *Arabidopsis*.

In this study, we test the editing efficiencies of CRISPR/LbCas12a driven by different Pol II promoters and different crRNA expression strategies in *Arabidopsis*. Our data showed that the editing efficiencies were greatly affected by different crRNA expression strategies and different promoters. The tRNA-crRNA fusion strategy was efficiently and precisely processed into crRNA and thus improved the editing efficiency. Furthermore, under the tRNA-crRNA fusion strategy, CRISPR/LbCas12a driven by RPS5Ap had the highest editing efficiency. Although the editing efficiency of CRISPR/LbCas12a system driven by EC1f-in(crR)p was low, the ratio of homozygous & bi‐allelic mutants was highest. Our results will further improve the editing efficiency of CRISPR/LbCas12a system and promote its application in *Arabidopsis*.

## Materials and methods

### Vector construction

The synthetic pNX-G and pUC57-LbCas12a were digested by *Xba*I *and Sac*I and allowed them ligated, resulting in the generation of pH-LbC. We amplified the *ApR* fragment from synthetic 212t-A with primers U6-AvF/tGlyLb-R0/tGlyLb-BbR and BB-ApF/U6-ESR, named U6Lb, AH, respectively. We mixed the pCBC-RPS5Am2 and pH-LbC, digested them with *Nco*I and *Xba*I, resulting in the generation of pHRLbC. The pH-RLbC and pCBC-U6Lb mixtures were digested by *Xba*I *and Sac*I, resulting in the generation of pHRLbA (tRNA-crRNA fusion strategy).

We replaced *Asc*I-*Pac*I fragment of synthetic pUC57-Csy4P2A with the *Sgr*AI/*Sac*I/*Bsa*I fragment of synthetic pUC57-ZsGreen, resulting in the generation of pUC57-Csy4ZsG. We amplified the pCBC-sgRC4 with primers Csy4T-BsF/F0@B2 and sgRC4-BsR, and allowed an insert, generated by annealing two oligos oC4T-F/R@B2, ligated with *Bsa*I-digested pL2L1-R5Csy4pA, resulting in the generation of pL2L1R-2xB21. We amplified two PCR fragments from pHRLbA with primers C4A-BKF/C4Lb-F0/hAp-BbR and hAp-BbF/AC4-R0/C4-AvR, respectively. We mixed the two fragments, purified them, digested them with KpnI/BbsI/AvrII, and allowed them ligated with *Kpn*I and *Avr*II-digested pL2L1R-2xB21, resulting in the generation of pL2L1-R5Lb2. We digested pL2L1-R5Lb2 with *Avr*II and *Spe*I, resulting in the generation of pHRLb-mChe. We digested pHRLb-mChe with *Sac*I and *Eco*RI, and allowed them ligated with pL2L1-R5Lb1 digested with *Sac*I and *Eco*RI, resulting in the generation of pHRLb-STU1 (STU).

We amplified different promoters by PCR, and we purifed the 4 PCR fragments, EC1f-U10, UBQ10, EC1f, YAO and set up the Gibson Assembly reaction, resulting in the generation of pHI-LbU6A (EC1f-in(crR)), pHE-LbU6A (EC1f), pHU-LbU6A (UBQ10), pHY-LbU6A (YAO). The primers used in this study were shown at S2 Table.

### Plant material and growth conditions

*Arabidopsis* ecotype Col-0 was used as the wild type. These plants grow on Murashige and Skoog (MS) media containing 0.8% Agar and 2% sugar. The seeds were treated at 4°C for 3 days, under 16 h light/8 h dark photoperiod for 1 week, and the plants of *Arabidopsis* were maintained in the greenhouse at 22°C.

### *Agrobacterium*-mediated transformation and analysis of mutations

We transformed 8 final LbCas12a binary vectors harboring one cassettes into *Agrobacterium* strain GV3101, Col-0 wild-type plants were used for *Agrobacterium*-mediated transformation via the floral dip method. To study the mutation of the target in more detail, genomic DNA was isolated from T1 transgenic plants and fragments with specific primers containing the target sites were amplified by PCR. Target GL1-M/N was amplified with primers Lb-F and Lb-R.

## Results

### Editing efficiencies of different crRNA expression strategies

**Fig 1:**
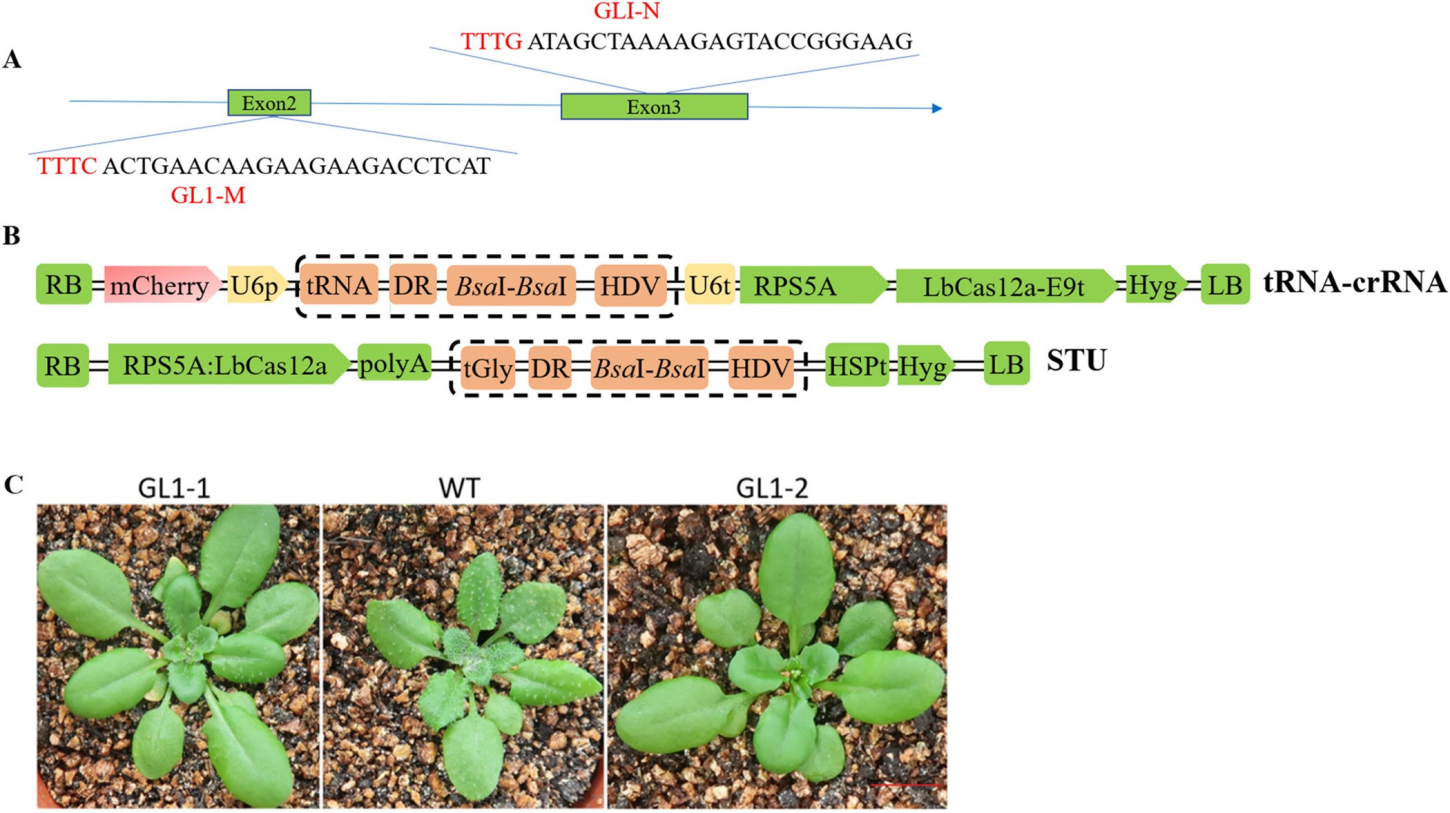
Editing efficiencies of different crRNA expression strategies. (A) The two target sequences of GL1-M and GL1-N. (B) Schematic diagram of T-DNA structure of different crRNA expression strategies. RB and LB, T-DNA right and left borders, re-spectively. U6p, *Arabidopsis U6* gene promoter. E9t, pea (Pisum sativum) rbcS-E9 terminators. HSPt, *Arabidopsis* HSP18.2 terminators. (C) Phenotypes of representative T1 mutant plants with tRNA-crRNA fusion strategy. GL1-1, partial glabrousness. WT, wild type. GL1-2, complete glabrousness. The length of the red line is 1cm.

In order to explore the most efficient way of expressing crRNA, we used two strategies to express crRNA. The first was based on the tRNA-crRNA fusion strategy and HDV ribozyme-based RNA processing systems [19]; The second was that co-expressing crRNA and LbCas12a in a single transcriptional unit (STU) provided a conditional, simple and highly active system for plant genome editing [20]. The structure of the vectors was shown in Fig 1B. To count editing efficiency easily, we chose the *GL1* gene as the target for efficient visual screen [21, 22], and designed two different target sites by using website (http://crispor.tefor.net/), named GL1-M and GL1-N (Fig 1A). The different CRISPR binary vectors were used to transform *Arabidopsis* Col-0 plants using the *Agrobacterium*-mediated floral dip method and a large number T1 transgenic lines were obtained. The editing efficiencies of different crRNA expression strategies were compared by next-generation sequencing. As for STU strategy, all target was not successfully edited in transgenic plants (GL1-M:0/189 and GL1-N: 0/162). In addition, we found two phenotype in T1 generation transgenic lines transformed with tRNA-crRNA fusion strategy: partial glabrousness and complete glabrousness (Fig 1C). A high mutation frequency of both targets was detected in the plants with tRNA-crRNA fusion strategy. The editing efficiencies were 65.53% (135/206) and 17.42% (31/178) for GL1-M and GL1-N, and the ratios of homozygous & bi‐allelic mutants were 2.0% (4/206) and 0.0% (0/178), respectively.

### Editing efficiency of CRISPR/LbCas12a driven by different promoters

**Fig 2:**
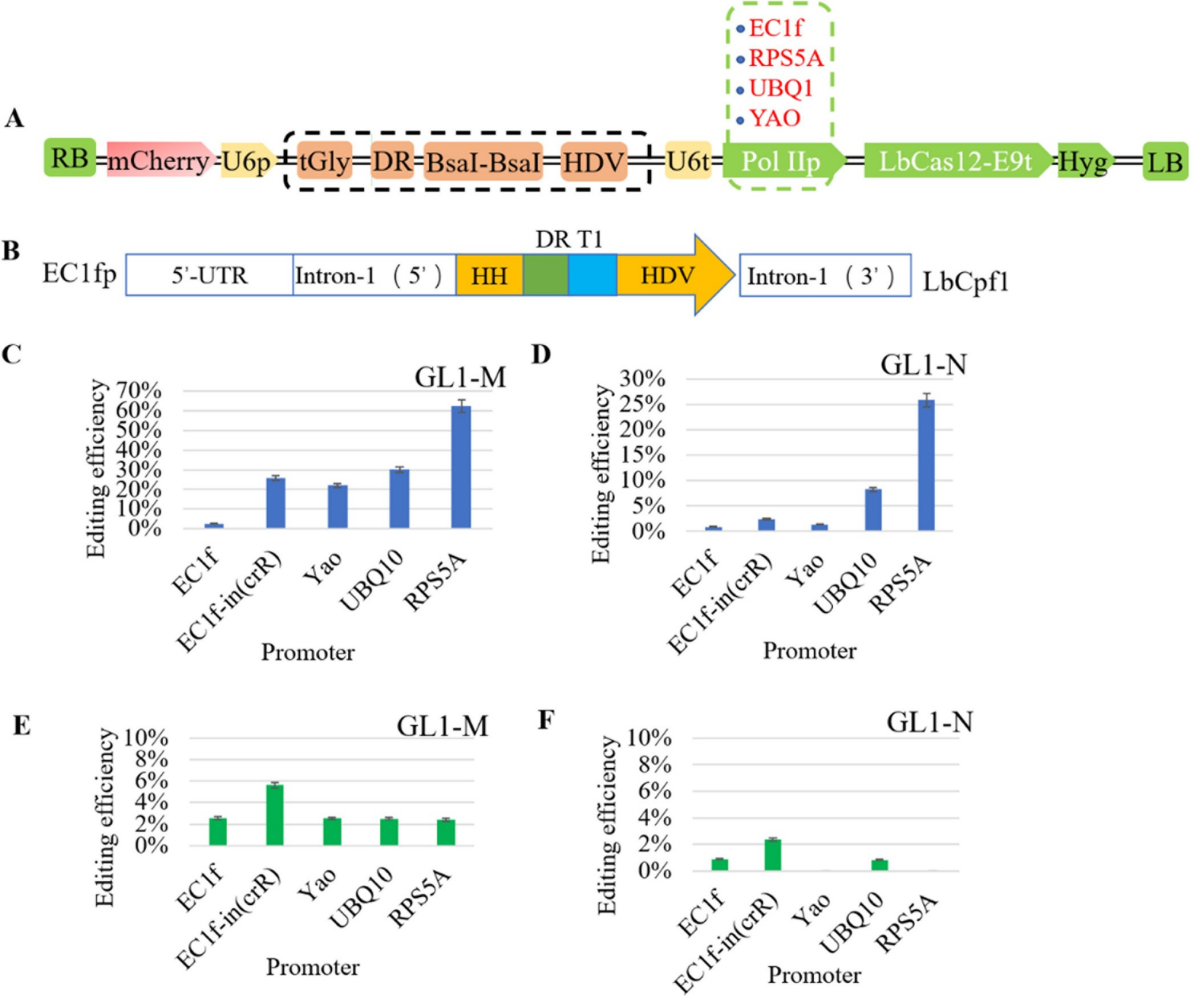
Editing efficiencies of CRISPR/LbCas12a driven by different promoters. (A) Schematic diagram of vector of the CRISPR/LbCas12a driven by different promoters. Green dotted line boxes includes the EC1fp, RPS5Ap, UBQ10p, and YAOp. (B) Schematic diagram of fusion promoter. (C) and (D) Editing efficiencies of different promoters in target GL1-M and GL1-N. (E) and (F) The rate of homozygous/bi-allelic mutants in target GL1-M and GL1-N.

Based on the expression cassette of tRNA-crRNA-HDV, the vectors of the LbCas12a driven by different promoters, i.e., EC1f [23], EC1f-in (crR), YAOp [24], UBQ10p [25] and RPS5Ap [17] were constructed and used to edit the *GL1* gene (GL1-M/N) of *Arabidopsis* (Fig 2A). EC1f-in (crR) was a novel fusion promoter that were constructed by inserting the UBQ10p intron between EC1fp and Cpf1, and the crRNA expression cassette was introduced into the intron (Fig 2B). The editing efficiencies of vectors with different promoters were compared according to the next-generation sequencing.

As for target GL1-M, the CRISPR/LbCas12a system driven by RPS5Ap had the highest editing efficiency, with a total mutation rate of 62.40%. Followed by UBQ10p, the total editing efficiency was 30.00%. The editing efficiency of CRISPR/LbCas12a driven by EC1fp was lowest, i.e., 2.56%. Moreover, we observed that the fusion promoter EC1f-in (crR) offered a 10-fold higher editing efficiency (25.84%) than that of EC1fp (Fig 2C and S1 Table). The editing efficiency of the target GL1-N was generally lower compared to that of the target GL1-M. The editing efficiency of vector driven by RPS5Ap was highest and followed by UBQ10p, with 25.84% and 8.26%, respectively, while that of EC1fp was lowest, i.e., 0.89% (Fig 2D and S1 Table).

### The ratio of homozygous & bi‐allelic mutants generated by different promoters in the T1 generation

The ratio of homozygous & bi‐allelic mutants generated by different promoters in the T1 generation was also evaluated. The results showed that the homozygous & bi‐allelic editing efficiencies of different promoters could be described in decreasing order as follows: EC1f-in (crR)>EC1fp>UBQ10p>YAOp>RPS5Ap (Fig 2E and 2F). Although the total editing efficiencies of EC1f-in (crR) and EC1fp were low, the rates of homozygous/bi-allelic mutants of these promoters were the highest, which were more conducive to obtaining heritable mutant in next generations. The fusion promoter of EC1f-in (crR) showed a significant advantage in producing homozygous/bi-allelic mutants.

### Analysis of heritable mutation in T2 generation

In order to obtain non-transgenic edited T2 generation lines more easily, we introduced a mcherry cassette driven by seed coat specific promoter AT2S3 into CRISPR/LbCas12a system for simple and reliable isolation of T-DNA free *Arabidopsis* mutants visually identified using a microscope [26]. Transgenic seeds had strong red fluorescence under microscope, while T-DNA free seeds had no light. We selected the non-red fluorescent seeds (T-DNA free seeds) under fluorescence stereoscopic microscope and seeded them. Four T1 lines of vectors with EC1f-in (crR)p (#1, #2, #3 and #4) and RPS5Ap (#5, #6, #7 and #8) were selected respectively to analyze the heritable mutant in T2 generation. We found that all lines can be stably inherited to the next generation and have no phenotypic separation. The detailed statistics were shown in the Table 1. This result indicated that the CRISPR/LbCas12a system driven by EC1f-in (crR) and RPS5Ap can stably inherit to the next generation.

**Table 1:** Analysis of transmittable mutants in T2 generation.

Line	Type of T1	Ratio of phenotype
#1	Ho	3/3 (100%)
#2	Bi	6/6 (100%)
#3	Ho	10/10 (100%)
#4	Bi	9/9 (100%)
#5	Ho	26/26 (100%)
#6	Bi	20/20 (100%)
#7	Bi	32/32 (100%)
#8	Ho	19/19 (100%)

Ho, homozygous; Bi, bi-allelic.

## Discussion

The CRISPR/LbCas12a system is an important genomic engineering tool for modifying target DNA sequences including gene knockout and fragment insertion in a variety of organisms. Although the system has been used in some plant species, including *Arabidopsis*. The editing efficiencies of CRISPR/LbCas12a system varied greatly between different plant species and tissues [20, 27, 28]. The editing efficiency of *Arabidopsis* under normal growth temperature (e.g., 22°C) was low [13]. Although the temperature-resistant CRISPR/enLbCas12a and CRISPR/ttLbCas12a system has been created recently to improve the editing efficiency at normal *Arabidopsis* growth temperatures. There is no report on improving the editing efficiency of the CRISPR/LbCas12a system by selecting the optimal crRNA expression strategies and promoter of LbCas12a in *Arabidopsis*. The results of this study filled the gap.

In this study, we conducted the comprehensive evaluation of the editing efficiency CRISPR/LbCas12a system that driven by different promoters of LbCas12a and crRNA expression strategies. The results showed that the tRNA-crRNA fusion strategy was more suitable for the expression of crRNA compared to the STU strategy. It may be result from that the tRNA-crRNA fusion strategy may be more conducive to crRNA mRNA transcription. In addition, neither target was successfully edited by using the STU strategy in all transgenic plants, which was inconsistent with the previous report that the STU strategy could improve the efficiency of gene editing in rice [29]. In this study, we conducted the comprehensive evaluation of the editing efficiency CRISPR/LbCas12a system that driven by different promoters of LbCas12a and crRNA expression strategies. The results showed that the tRNA-crRNA fusion strategy was more suitable for the expression of crRNA compared to the STU strategy. It may be result from that the tRNA-crRNA fusion strategy may be more conducive to crRNA mRNA transcription. In addition, neither target was successfully edited by using the STU strategy in all transgenic plants, which was inconsistent with the previous report that the STU strategy could improve the efficiency of gene editing in rice [13]. Based on the expression cassette of tRNA-crRNA-HDV, different promoters were used to drive LbCas12a in CRISPR/LbCas12a system. We found that the CRISPR/LbCas12a system driven by the RPS5Ap had high editing efficiency for the all targets, which may be result from that the RPS5A promoter maintains high constitutive expression at all developmental stages [17]. Although the editing efficiencies of EC1fp and EC1f-in(crR)p in the T1 generation was low, the percentage of homozygous/bi-allelic mutants was higher than that of other promoters, which was related to the specific expression characteristics of this promoter in egg cells and single-cell stage embryos [23].

Moreover, the promoter of EC1f-in(crR) was constructed by introducing UBQ10 introns into the EC1fp and the editing efficiency was greatly improved compared to the that of EC1fp. Therefore, we concluded that the introduction of introns in EC1fp could improve the editing efficiency of CRISPR/LbCas12a system due to the increased expression level of LbCas12a, which could provide a reference for improving the editing efficiency of other gene editing systems. Interestingly, we also found that the editing efficiency of the same promoter differed significantly between the two target sites in the GL1-M/N, indicating that the CRISPR/LbCas12a has a high selectivity for the target [27–29].

In conclusion, the results of our study suggested that tRNA-crRNA fusion strategy was more suitable for the expression of crRNA and the CRISPR/LbCas12a driven by RPS5Ap has the highest editing efficiency, while EC1f-in (crR)p has the highest ratio of homozygous & bi‐allelic mutants. The results of this study will improve the editing efficiency of CRISPR/LbCas12a system, thus promoting its application in *Arabidopsis* and prove useful for optimization of CRISPR/LbCas12a methods in other plants.

## Supporting information

S1 Table Editing efficiencies of different promoters. (DOCX)

S2 Table Primers used in this study. (DOCX)
