# Supplementary material for: Optimization of CRISPR/LbCas12a-mediated gene editing in Arabidopsis
Source: PLoS One. 2022 Mar 25;17(3):e0265114. doi: 10.1371/journal.pone.0265114 (PMC8956186; doi:10.1371/journal.pone.0265114)
Supplement: S1 Table — (DOCX) [file pone.0265114.s001.docx]

# S1 Table Editing efficiency of different promoters

| Genome target | Promoter | NO.of edited plans | NO.of Ho/Bi mutant | NO.of plants identified | Mutation rate (%) | Ho/Bi mutant rate (%) |
| --- | --- | --- | --- | --- | --- | --- |
| GL-M | EC1f | 2 | 2 | 78 | 2.56% | 2.56% |
|  | EC1f-in(crR) | 23 | 5 | 89 | 25.84% | 5.62% |
|  | YAO | 35 | 3 | 159 | 22.01% | 1.89% |
|  | UBQ10 | 36 | 3 | 120 | 30.00% | 2.50% |
|  | RPS5A | 78 | 3 | 125 | 62.40% | 2.40% |
| GL-N | EC1f | 1 | 1 | 112 | 0.89% | 0.89% |
|  | EC1f-in(crR) | 2 | 2 | 84 | 2.38% | 2.38% |
|  | YAO | 2 | 0 | 145 | 1.38% | 0.00% |
|  | UBQ10 | 10 | 1 | 121 | 8.26% | 0.83% |
|  | RPS5A | 23 | 0 | 89 | 25.84% | 0.00% |

Ho, homozygous; Bi, bi-allelic.
