# Supplementary material for: Optimization of CRISPR/LbCas12a-mediated gene editing in Arabidopsis
Source: PLoS One. 2022 Mar 25;17(3):e0265114. doi: 10.1371/journal.pone.0265114 (PMC8956186; doi:10.1371/journal.pone.0265114)
Supplement: S2 Table — (DOCX) [file pone.0265114.s002.docx]

**S2 Table Primers used in this study**

| Primer | Sequence |
| --- | --- |
| U6-AVF | ATTTATTTTACCTAGGCGACTTGCCTTCCGCAC |
| TGLYLB-R0 | ACATCTACACTTAGTAGAAATTATGCACCAGCCGGGAATC |
| TGLYLB-BBR | ACATATTTGAAGACACATCTACACTTAGTAGAAATTATGC |
| LBCPF1-IDF | CGACTTCTTCAAGGACTCCATC |
| LBCPF1-IDR | GTAGGAGAGGGTCGTGGTCTTC |
| BB-APF | ATATTTGAAGACGTAGATAGAGACCTTCGGGGAAATGT |
| EC1.2EN-IDF3 | TCCCATTCCTCCCACTAATCCAACT |
| EC1.1P-IDR | ATTAGAATCACTCAGTCTGAAACTCGTT |
| CSY4T-BSF@B2 | ATTTATTGGTCTCTGCAGCAAGCAATAAGCTCGATGGG |
| CSY4T-F0@B2 | CAAGCAATAAGCTCGATGGGGTTTTAGAGCTAGAAATAGC |
| SGRC4-BSR | ATTATTTGGTCTCTGAACGCACCGACTCGGTGCCACTT |
| OC4T-F@B2 | GTTCACTGCCGTATAGGCAGCAAGCAATAAGCTCGATGGG |
| OC4T-R@B2 | AAACCCCATCGAGCTTATTGCTTGCTGCCTATACGGCAGT |
| U6P2-KPNI | ATTATTACGGTACCAAGTTGAAAACAATCTTCAAAAGTCC |
| U6T3-AVR2 | ACATTTATTACCTAGGAAGAAGAAATCG |
| HAP-BBR | ATATTTATGAAGACTGGTCCTCCGATCGTTGTC |
| HAP-BBF | ATTTATTTGAAGACATGGACCGAAGGAGCTAACCGCTT |
| C4A-BKF | ATTATTGAAGACATAATTGGTACCGTTCACTGCCGTATAG |
| C4LB-F0 | GTTCACTGCCGTATAGGCAGTAATTTCTACTAAGTGTAGA |
| AC4-R0 | CTGCCTATACGGCAGTGAACTGAGACCTTGGTCATGGGATTATC |
| C4-AVR | ATTATTACCTAGGCTGCCTATACGGCAGTGAAC |
| Lb-F | ATAGATACAATTAAACCAAC |
| Lb-R | TAACTAACTTATTTTTCACA |
